# Enhancing Policy Impact Through Knowledge Translation: The Role of Innovative Evidence Products

**DOI:** 10.1002/cl2.70084

**Published:** 2025-12-01

**Authors:** Ashima Mohan, Howard White

**Affiliations:** ^1^ Campbell South Asia New Delhi India; ^2^ The Research and Evaluation Centre London UK

## 1 Introduction

The objective of applied social science research is to inform policy and practice to improve societal outcomes (White and Welch 2022). However, in the words of the head of the World Bank's Development Impact Group, DIME, ‘Dissemination is dead’.^1^ That is, traditional dissemination pathways—academic publications, conference presentations, and even policy briefs—are insufficient for achieving meaningful policy uptake. No matter how rigorous or relevant, research alone does not usually translate into policy impact without deliberate, structured mechanisms to bridge the gap between evidence generation and decision‐making. The recognition of this fact has resulted in the growth of knowledge translation as what has been called the fourth wave of the evidence revolution (White 2019).

Knowledge translation is ‘the exchange, synthesis, and effective communication of reliable and relevant research results. The focus is on promoting interaction among the producers and users of research, removing the barriers to research use, and tailoring information to different target audiences so that effective interventions are used more widely’ (World Health Organization 2004). There are various approaches to knowledge brokering. These include direct interaction between researchers and decision‐makers in interpreting and using the findings, in‐house knowledge brokers, creating a ‘helpdesk’ function, or using an independent rapid review service.

The approach we discuss here is online evidence‐based decision‐making products (EBDMPs), which summarize research findings in accessible forms without requiring the user to read the underlying research papers or reports. The traditional policy brief is an example of an EBDMP. However, a policy brief alone is usually insufficient to engage decision‐makers.

Policy briefs have been the cornerstone of knowledge translation for many years. In 2015, the Campbell Collaboration began to publish Plain Language Summaries (PLSs) of new reviews, as well as producing PLSs for most existing reviews.^2^ The co‐chair of the Crime and Justice group described these PLS as ‘gold dust’.^3^ Whilst policy briefs alone will have a limited impact on decision‐makers, the value placed by the Campbell Crime and Justice co‐chair on the PLS was because he used them as part of his engagement with high‐level decision‐makers, not as a standalone product.

The shortcomings of policy briefs include that (i) they are uni‐directional communication of researchers telling decision‐makers what to do, rather than engaging them as stakeholders in the research process; (ii) policy briefs of single studies may have limited discussion of context and transferability of study findings, and (iii) lack comparisons to alternative approaches. In addition, there is often a focus on ‘what works’, but with insufficient information on intervention design and implementation to be of use.

Hence, more sophisticated EBDMPs are needed than a policy brief. These have been classified as evidence portals, guidelines, and checklists (White 2019). Evidence portals are our focus here.

## 2 Evidence Portals: An Emerging Model

Evidence portals provide interactive, web‐based platforms that curate and categorize evidence in user‐friendly formats. The first examples come from the education sector, starting with the US Institute of Education Science's What Works Clearinghouse, and the Education Endowment Foundation's (EEF) Teaching and Learning Toolkit.^4^ The EEF toolkit lists over 30 approaches to improving learning outcomes, such as arts participation, feedback, peer tutoring and repeating a year. For each intervention, the toolkit landing page reports three key metrics: effect size (impact magnitude), cost rating, and strength of evidence. The impact is the months of additional learning outcomes for children exposed to the intervention compared to comparable children not exposed to the intervention. This impact is calculated through a statistical meta‐analysis. Cost and evidence strength are reported on a five‐point scale. For example, providing children feedback on their work is very low cost and has an impact of 6 months additional learning, based on a strong evidence base (4 out of 5).

The toolkit is used by over 70% of secondary schools in England and Wales. This means that 70% of schools are making decisions about what to do based on evidence from systematic reviews. This is not because teachers have read these systematic reviews, or even know what a systematic review is, but because the toolkit is a heavily curated evidence product, making review evidence available in an accessible form, and one that allows comparison of many different approaches in a single place.

### 2.1 The Youth Endowment Fund (YEF) Toolkit

We describe here in more detail the similar toolkit of the YEF on strategies for reducing youth offending, which we worked on under the auspices of the Campbell Collaboration.

As a first step, the Campbell team produced an evidence and gap map (EGM) of interventions to reduce youth involvement in crime and violence.^5^ EGMs should not usually be seen as an end in themselves, but as an instrumental product to help build the evidence architecture (White 2021). Specifically, they can identify existing reviews suitable for use to develop toolkit content, clusters of unreviewed primary studies in areas of interest, and areas in which primary studies are needed. This YEF map contained over 2000 studies, including over 200 systematic reviews. The first edition of the toolkit was published with 17 approaches, based on a combination of the availability of recent systematic reviews identified in the EGM which contained a meta‐analysis of relevant outcomes, and YEF's stakeholder consultation on priorities.

Later additions added approaches from reviews undertaken by Campbell or those which Campbell commissioned on behalf of YEF. Specifically, teams led by Campbell South Asia staff conducted three in‐house reviews (mentoring, Lakshminarayanan et al. 2022; adventure and wilderness therapy, Mohan et al. 2022; and sports, Malhotra et al. 2022). Commissioned reviews include arts participation (Mansfield et al. 2024), post‐custody programme (Wong et al. 2023), and stop and search (Petersen et al. 2023).

Following the format of the EEF toolkit, the YEF toolkit landing page presents three metrics:

1. Impact, divided into harmful, no effect, low, moderate and high, where these categories are based on Cohen's *d* effect size.
2. Evidence strength rating based on the size of the evidence base, critical appraisal of the review, and heterogeneity. Ideally, evidence strength would have also been based on confidence in the included studies in the review, but this was not reported in all reviews, and so it was not possible.
3. The cost, which was collected by YEF from UK practitioner agencies.

After just one year, the toolkit had already had considerable influence on decision‐makers including the Home Office requiring Violence Reduction Units to spend 25% (later raised to 30%) of their budget on interventions shown to be effective by the toolkit, local authorities using the toolkit to revise their youth violence strategies, and the head of YEF using the toolkit in conversation with the Prime Minister's Office to persuade them to abandon a proposed bootcamp programme in favour of focused deterrence.^6^

### 2.2 Youth Employment in Sub‐Saharan Africa Evidence Products

A second toolkit produced by the Campbell team covers interventions for youth employment in sub‐Saharan Africa. This project, supported by the European Commission, started by updating the youth employment EGM, first produced for the Mastercard Foundation, and previously updated for the Youth Futures Foundation (Apunyo et al. 2022). The work on the EGM has been mainly undertaken by a team from the Africa Centre for Systematic Reviews and Knowledge Translation, Makerere University, Uganda, under the guidance of the former Campbell CEO.

Through a consultation with the EC, ILO, the World Bank and selected African policy makers, a list of 10 interventions was chosen. The team carried out an overall meta‐analysis, including a component network meta‐analysis, as well as intervention‐specific meta‐analyses for each of the 10 interventions. We also produced a qualitative synthesis report. Technical reports were produced for each of the 10 interventions. These findings were combined in a mixed‐methods review (White et al. 2020).

The toolkit landing page adopts the usual metrics of impact on employment, evidence strength and cost of the intervention. It also reports the impact on skills, earnings or business development where this effect is relevant and available. As in other toolkits, the second level gives a more detailed discussion of each intervention, including a section on design choices.

The EC website contains three products^7^: the EGM, the toolkit, and the mixed method synthesis, which was prepared as a standalone document, providing summary evidence across all interventions.

## 3 Evidence Q&A: A Structured Approach to Evidence Accessibility and Use

Like the evidence portals discussed above, in the Evidence Q&A for Gender and Agriculture in a Changing Climate, produced by Campbell South Asia, all content is based on systematic reviews.^8^ The portal uses a layered question‐and‐answer structure. Users begin at the top level with seven broad, policy‐relevant questions, such as ‘How are women farmers in rural areas adapting to climate change?’ From there, they can drill down into more specific sub‐questions—typically between four and eight per topic—that narrow the focus to particular mechanisms or practices, such as the role of traditional knowledge in adaptation.

Each of these sub‐questions is supported by a three‐part content structure. A short summary, a long summary, and a technical review digest that links directly to the systematic reviews themselves, along with review‐level summaries. In addition to the question‐and‐answer format, each of the seven main questions is linked to a one‐page policy brief. The CGIAR Evidence Q&A platform will allow for regular updates as new systematic reviews become available and allows for expansion into new thematic areas as policy needs shift.

To help explore the evidence, the platform has filters that allow users to navigate the evidence. Content is tagged with themes such as adaptation (covering behavioural, technological, and infrastructural responses), mitigation (strategies for reducing emissions and managing carbon), innovation (emerging gender‐sensitive technologies and delivery methods), governance (institutional structures and decision‐making), and livelihoods (including income, assets, and safety nets). This means, for example, that a user interested in rural women's adaptation strategies can quickly identify relevant evidence within the broader governance context.

The CGIAR evidence Q&A platform is a good practice example of translating research into usable policy guidance. Its structure reflects three core insights. First, that decision‐makers do not need more data—they need reliable, well‐organized evidence that directly addresses the questions they are facing. Second, systematic reviews provide the necessary credibility, but thoughtful design and navigation are what make the evidence accessible. And, third, that integrating gender, agriculture, and climate within a single knowledge product mirrors the complexity of real‐world development challenges, enabling more coherent and coordinated action.

## 4 Putting the Evidence in EGMs: Mapping the Landscape

The final approach we discuss here is ‘putting the evidence in evidence and gap maps’, by which we mean a map with cell‐wise evidence summaries.

EGMs, pioneered by 3ie and further developed by the Campbell Collaboration, provide an interactive visualization of the distribution and strength of evidence across thematic and methodological dimensions (White et al. 2020). Maps allow users to both navigate and access the evidence. However, they do not tell users what the evidence says.

The Campbell team produced a version of the EGM of interventions in institutional settings to address child maltreatment (Finch et al. 2021), which summarized the evidence in each cell. In a usual EGM, clicking a cell allows the user to view and access the list of studies in that cell. In the child protection EGM, the top entry in that list is a short version of the cell‐level summary providing the main findings on effectiveness. At the end of the short summary is a link to a PDF of the long summary.

The Child Protection Research website, where this EGM is available,^9^ also includes various different presentations of the evidence under the headings ‘What Works’ (classified as what works, what we do not know, and evidence gaps), and ‘Finding Evidence’, which provides very brief summaries for each intervention sub‐category. This content is based on the Guidebook the team produced, which combines the contents of a usual descriptive EGM report (what the studies are about, where they are from, etc.) with summaries of what the evidence says. The site, primarily the Guidebook, has been used extensively by the funder to make programming decisions.

We are producing a second EGM—on conflict and atrocity prevention (CAP)—where we are putting the evidence in the EGM. There are two differences with the child protection EGM. First, there is the number of studies and cells needing to be summarized. The child protection map had only 136 studies compared to 573 in the CAP EGM, with over 250 cells to be summarized. Second, in the CAP EGM, we are producing cell‐wise effect sizes. This has involved effect size extraction and calculation of the cell‐wise average effect for which we used the Shinyapp produced by Josh Polanin and colleagues.^10^

## 5 Lessons Learned From Developing Evidence Products

Experience from the design and deployment of evidence platforms and related tools has yielded several important lessons that inform effective knowledge translation practices.

The first is to illustrate that both systematic reviews and EGMs can be used as the basis for a range of evidence products. This is the process of knowledge translation, turning one type of evidence product (maps and reviews) into an EBDMP that allows the user to access the evidence.

The second lesson is the importance of transparent evidence standards and methodological rigour. The credibility of any evidence product depends on the transparency and robustness of the methods used to generate it. These are necessary for the product to be trusted. This includes clear inclusion criteria, systematic quality appraisal processes, and transparent synthesis techniques. For example, the technical manual developed for the YEF toolkit (YEF 2025) outlines precise criteria for evaluating the relevance, design quality, and generalizability of evidence, as well as how to select the estimate of impact, thus helping to ensure that users can rely on the information provided to guide decisions.

Also important is accessibility and ease of use. Even the most rigorous evidence has limited value if it is not usable. Effective evidence products prioritize simplicity and clarity in both content and design. This includes the use of visual summaries, tiered content structures, and standardized formats that enable users to navigate complex information quickly and efficiently. The most successful platforms are those that offer intuitive interfaces and are optimized for use across devices, including mobile phones and tablets, allowing access in varied operational environments.

Moreover, EBDMPs are most effective when they are not standalone tools but part of a broader suite of complementary products. These may include implementation guidelines, logic models or theories of change, cost calculators, and practice handbooks based on process evaluations. Each of these plays a distinct role in bridging the gap between knowing what works and applying that knowledge in real‐world settings. In particular, guidelines are important to ensure fidelity during implementation and to support scaling efforts by offering concrete, actionable steps grounded in the available evidence.

The final important lesson is the importance of stakeholder co‐production. Evidence products have the greatest impact when they are developed in close collaboration with those who will use them—policymakers, practitioners, and community‐based organizations. Engaging end‐users from the outset ensures that the questions addressed are grounded in practical realities and that the products themselves are aligned with policy priorities. This kind of participatory process strengthens both the relevance and the legitimacy of the resulting tools.

However, these approaches are not without their critics. While support for knowledge translation has grown in recent years, many academic researchers remain hesitant to participate directly in the development of applied evidence products. Criticism from within academia often centres on concerns about oversimplification, methodological compromises, or insufficient theoretical grounding. Researchers from a constructivist perspective argue that the limits to transferability undermine the usefulness of EBDMPs. One response to these concerns is the methodological rigour incorporated in publicly available evidence standards to enhance transparency. While it will not satisfy all critics, EDMPs should enable the user to look beyond average effects to understand sources of heterogeneity.

## 6 Conclusion

Improving public policy outcomes demands more than generating knowledge—it requires translating it into usable formats. EBDMPs such as those developed with CGIAR, YEF, and others demonstrate how structured, digital tools can significantly enhance the uptake of research. The key is designing products with a focus on clarity, credibility, and usability, while maintaining rigorous evidence standards.

As demand grows for evidence‐informed decision‐making, these tools will play a vital role in shaping policy ecosystems that are responsive, inclusive, and data‐driven. We expect there to be a growing number and range of EBDMPs in the coming years, and hope that this short editorial contributes to their development.

## Endnotes

1 Presentation by Arrianne Legovini to Network of Networks for Impact Evaluation (NONIE) meeting, Paris, 2008.

2 A limited number of Policy Briefs were published covering a set of reviews on common themes. These were agricultural development, early child development, and policing.

3 Personal communication from Peter Neyroud.

4 See https://eef.dev/education-evidence/teaching-learning-toolkit.

5 See https://youthendowmentfund.org.uk/evidence-and-gap-maps/programmes-evidence-and-gap-map/.

6 Presentation by Peter Henderson, YEF, on ‘Uses of YEF Toolkit’, June 2022.

7
https://capacity4dev.europa.eu/resources/Youth-Employment-Sub-Saharan-Africa-Toolkit_en.

^8^

https://mediumorchid-mule-525077.hostingersite.com/.

9
https://child-protection-research.org/.

10
https://airshinyapps.shinyapps.io/mosaic_egm/.
